# Single Particle and PET-based Platform for Identifying Optimal Plasmonic Nano-Heaters for Photothermal Cancer Therapy

**DOI:** 10.1038/srep30076

**Published:** 2016-08-02

**Authors:** Jesper Tranekjær Jørgensen, Kamilla Norregaard, Pengfei Tian, Poul Martin Bendix, Andreas Kjaer, Lene B. Oddershede

**Affiliations:** 1 Dept. of Clinical Physiology, Nuclear Medicine & PET and Cluster for Molecular Imaging, Rigshospitalet and University of Copenhagen, Denmark; 2 Niels Bohr Institute, University of Copenhagen, Denmark; 3 Laboratory of Chemical Physics, National Institute of Diabetes and Digestive and Kidney Diseases, National Institutes of Health, Bethesda, Maryland, United States

## Abstract

Plasmonic nanoparticle-based photothermal cancer therapy is a promising new tool to inflict localized and irreversible damage to tumor tissue by hyperthermia, without harming surrounding healthy tissue. We developed a single particle and positron emission tomography (PET)-based platform to quantitatively correlate the heat generation of plasmonic nanoparticles with their potential as cancer killing agents. *In vitro,* the heat generation and absorption cross-section of single irradiated nanoparticles were quantified using a temperature sensitive lipid-based assay and compared to their theoretically predicted photo-absorption. *In vivo,* the heat generation of irradiated nanoparticles was evaluated in human tumor xenografts in mice using 2-deoxy-2-[F-18]fluoro-D-glucose (^18^F-FDG) PET imaging. To validate the use of this platform, we quantified the photothermal efficiency of near infrared resonant silica-gold nanoshells (AuNSs) and benchmarked this against the heating of colloidal spherical, solid gold nanoparticles (AuNPs). As expected, both *in vitro* and *in vivo* the heat generation of the resonant AuNSs performed superior compared to the non-resonant AuNPs. Furthermore, the results showed that PET imaging could be reliably used to monitor early treatment response of photothermal treatment. This multidisciplinary approach provides a much needed platform to benchmark the emerging plethora of novel plasmonic nanoparticles for their potential for photothermal cancer therapy.

Photothermal therapy, which involves the application of plasmonic nanoparticles as light-triggered thermal transducers^1^,^2^,^3^,^4^,^5^, has emerged as a promising cancer treatment strategy within the last decade^6^,^7^,^8^,^9^, and much effort is being put into developing the most efficient light-to-heat converters^10^,^11^,^12^,^13^,^14^,^15^. The temperatures generated upon laser irradiation of plasmonic nanoparticles are so extreme^16^ that nearby cancer cells immediately ablate or become irreversibly damaged; meanwhile the heating is sufficiently localized that surrounding tissue is left unharmed^17^,^18^,^19^,^20^. The strongly enhanced photoabsorption properties of plasmonic nanoparticles are a consequence of a phenomenon known as surface plasmon resonance; the collective oscillations of the plasma electrons when resonant with the excitation light^21^. The absorption spectrum of a plasmonic nanoparticle can be tuned by changing the size, shape and composition of the nanoparticle^12^,^22^,^23^,^24^,^25^, and in particular near infrared (NIR) resonant nanoparticles are of interest for photothermal therapy due to the efficient penetration and low absorption of NIR light in tissue^26^. A second factor crucial for photothermal therapy is delivery of nanoparticles to the tumor. One of the great advantages of nanomedicine for cancer therapy is that nano-sized drugs and particles, when injected into the bloodstream, passively accumulate in tumor tissue through the leaky tumor vasculature^27^,^28^; a phenomenon known as the enhanced permeability and retention (EPR) effect. The EPR effect varies between tumor types^29^, but in general favors accumulation of sub-100 nm structures^30^. As a fraction of the nanoparticles will end up in healthy tissue after being injected intravenously, especially in the liver and spleen^31^,^32^, it is also important to consider the biocompatibility of the nanoparticles. For these reasons, focus has been put on identifying and developing plasmonic nanoparticles which are efficient light-to-heat converters, are biocompatible, accumulate efficiently by the EPR effect, and ultimately can minimize or eliminate tumors *in vivo*. Gold nanostructures meet these requirements and provide easy surface chemistry for modifications with functional biomolecules^3^. Spherical, solid gold nanoparticles (AuNPs) are easily synthesized, but in the size range useful for EPR-based tumor delivery their plasmon resonance peaks are in the visible region; hence, they may not be optimal candidates for *in vivo* photothermal therapy^19^,^24^. Silica-gold nanoshells (AuNSs)^33^, composite structures with a silica core surrounded by a thin gold shell, were the pioneering plasmonic nanoparticles used for photothermal therapy^6^,^7^,^34^. The AuNSs show great photothermal efficacy at NIR wavelengths, however, a relatively large ratio of scattering to absorption efficiency^35^, as well as diameters >100 nm which limit the EPR-based tumor delivery, somewhat lower their potential *in vivo*. Gold nanorods, which have efficient longitudinal mode absorption in the NIR even at sub-100 nm dimensions, have also been put forward as efficient light-to-heat converters and as candidates for photothermal therapy^24^,^36^,^37^. However, their potential is limited by their synthesis that involves high concentrations of cytotoxic surfactants that are difficult to remove entirely. Furthermore, nanorods are only efficient light-to-heat converters if they are aligned with the polarization vector of the irradiating laser light, and they have been shown to restructure into non-resonant shapes upon irradiation, even at relatively low intensities^38^. In recent years, new generations of multi-layered structures such as the nanomatryoshkas^15^,^39^, as well as more exotic shapes such as nanohexapods^12^,^14^, nanocages^9^,^40^ or nano-raspberries^10^ have been developed with the aim of optimizing NIR absorption efficiencies and having dimensions relevant for tumor retention *via* the EPR effect.

It has proved non-trivial to directly measure or theoretically predict the thermal response of irradiated nanoparticles, this is mainly because the light intensity and other physical parameters at the position of the nanoparticle are not known and the particle might disintegrate or change shape^38^ upon irradiation. Moreover, the understanding of how nanoparticles interact with electromagnetic radiation is not complete. Therefore, as additional types of optimized plasmonic nanoparticles are continuously being developed there is an urgent need for efficient strategies to quantify and compare their light-to-heat converting capabilities, and thereby their potential for photothermal cancer therapy.

Here, the heat generation of NIR-absorbing AuNSs was benchmarked against AuNPs that mainly absorb light in the visible region. *In vitro*, the nanoparticles were compared on a single particle level using an assay based on a temperature-sensitive biological matrix that allowed for a direct quantification of both the temperature profile of a single NIR irradiated nanoparticle and its absorption cross-section which can be used to predict the ability of the particle to heat macroscopic environments. To our knowledge, this is the first quantitative assessment of the temperature profile of a NIR irradiated single AuNS. *In vivo*, the nanoparticles were injected directly into subcutaneous human tumor xenografts in mice and the photothermal effect of the nanoparticles was quantified by PET imaging with a ^18^F-labelled glucose analogue, ^18^F-FDG. This provided a quantitative measure of decreased tumor metabolism in treated areas as early as an hour after treatment. The results of our *in vitro* evaluation of the nanoparticles are consistent with their *in vivo* therapeutic effect and we here demonstrate a strategy that can be reliably used to screen and evaluate the potential of any nanoparticle for photothermal treatment.

## Results

### Photo-absorption of plasmonic nanoparticles

**Figure 1:**
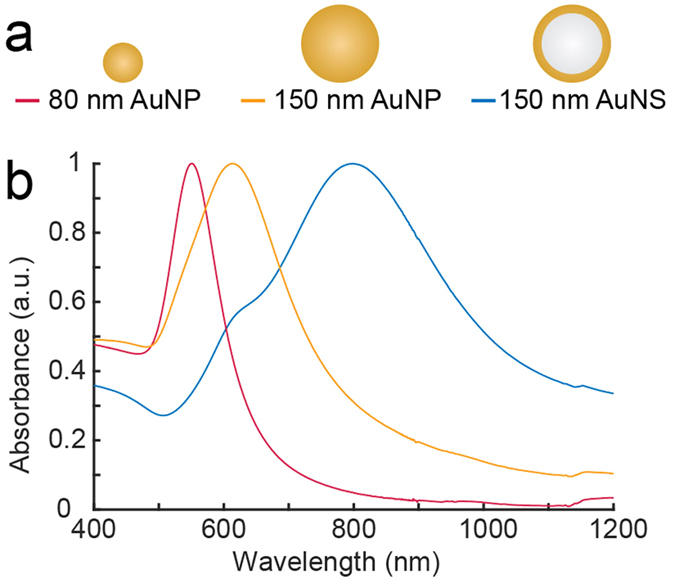
Characteristics of the plasmonic nanoparticles. (**a**) Sketch of the investigated plasmonic nanoparticles. (**b**) Absorption spectra in water for the three investigated nanoparticle types show that AuNPs with diameters of 80 nm and 150 nm exhibit resonances in the visible region (400–700 nm), whereas the AuNSs (diameter 150 nm) have their resonance peak in the near infrared region (700–1100 nm).

For this study we chose to compare nanoparticles with potential for photothermal cancer therapy using the criteria that the particles should be commercially available in a highly homogeneous quality, they should be biocompatible, and they should not restructure upon NIR irradiation. To probe the sensitivity of our methods, both nanoparticles resonant with NIR light and nanoparticles non-resonant with NIR light were tested. As a NIR resonant nanoparticle we selected AuNSs with a total diameter of 150 nm (core diameter = 120 nm, shell thickness = 15 nm, see Fig. 1a). These dimensions provide a peak absorption at 799 nm which overlaps well with the laser wavelengths used (807 nm for the *in vivo* experiments and 1064 nm for the single particle *in vitro* experiments; see Fig. 1b). To compare to a NIR non-resonant nanoparticle, we selected spherical solid AuNPs with the same diameter (150 nm) and peak absorption at 614 nm (see Fig. 1). Importantly, the NIR resonant AuNSs are considered too big for efficient EPR mediated tumor accumulation, and reducing their dimensions would imply shifting their resonance^33^,^41^. Therefore, we included a AuNP with a dimension more compatible with EPR accumulation in the study, namely a AuNP with a diameter of 80 nm^27^,^30^. Notably, 80 nm AuNPs are not NIR resonant (their peak absorption is at 550 nm; see Fig. 1b).

To theoretically compare the absorption of the nanoparticles we calculated the absorption cross-sections using the Finite Element Method (FEM) (see Supplementary Fig. S1). The absorption cross-section is directly related to the light-to-heat conversion efficiency of a nanoparticle and is therefore a suitable comparative theoretical measure^25^,^35^,^42^. We found that at the wavelength of the laser used for the *in vivo* experiment (807 nm) the absorption cross-section of the AuNSs was nearly 6 times larger than that of the 150 nm AuNPs with the same particle diameter, at 1064 nm the difference was a factor of 3. Furthermore, the absorption cross-section, which increases with volume, but only in a linear fashion as long as the radius is smaller than the skin depth^43^, was found to be an order of magnitude smaller for the 80 nm AuNPs compared to that of the 150 nm AuNPs.

### Quantifying single particle heating *in vitro*

To quantify the heat generation of a single irradiated nanoparticle *in vitro* we used the inverse relationship between the temperature increase of a nanoparticle, Δ*T*, and the distance, *D*, to the particle center described by the relation^44^:


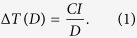


**Figure 2:**
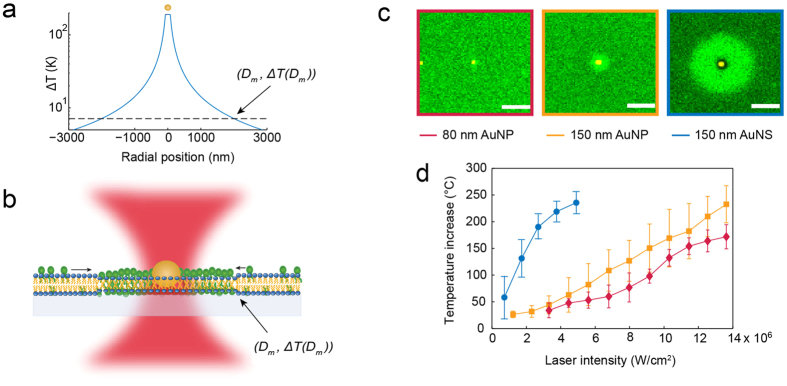
Measuring single particle heat generation using a 2D phospholipid bilayer assay. (**a**) The temperature profile of an irradiated AuNS (diameter 150 nm) at the laser intensity *P = *2.7 × 10^6^ W/cm^2^. The temperature decays with increasing distance from the particle center. (**b**) The biological assay used to map out the temperature profile. The assay consists of a 2D phospholipid bilayer with a well-characterized phase transition and a fluorescent molecule that senses the phase and preferentially partitions into the fluid phase. The surface temperature of the nanoparticle can be extracted by determining one point on the temperature profile, **Δ*****T***, in this assay corresponding to half the diameter of the melted footprint, ***D_m_***. (**c**) Optically heating a nanoparticle embedded in the bilayer with a tightly focused laser beam induces local melting. Here is shown representative melted footprints for all three nanoparticles at the same laser intensity, *P = *4.3 × 10^6^ W/cm^2^. The fluorescent images are recorded by acquiring an emission bandwidth of 496 nm–587 nm and the nanoparticles are imaged by recording 594 nm laser light reflected from the nanoparticle. Scale bars are 2 μm. (**d**) The surface temperature as a function of laser intensity for all three nanoparticles derived from the size of the melted footprints and using equation (1). *n* = 12 for all datasets and error bars represent one standard deviation (SD).

Here, *C* is a physical constant containing the thermal conductivity of water and glass, the particle radius and the absorption cross-section of the nanoparticle, and *I* is the intensity of the laser. If *C* can be extracted at one point on the nanoparticle’s temperature profile, equation (1) can be used to map out the entire temperature profile and the temperature increase at any distance from the nanoparticle–center, including the surface temperature, can be inferred (see Fig. 2a)^16^,^38^,^45^.

To measure the temperature at some distance from the irradiated nanoparticle we used an assay based on the well-characterized gel-to-fluid phase transition of a 2D phospholipid (DC_15_PC) bilayer (see Fig. 2b). Detection of the local heating around the irradiated nanoparticle was facilitated by incorporating fluorescent molecules with phase sensitive lipid anchors in the supported lipid bilayer^16^,^25^,^38^. A tightly focused Gaussian 1064 nm laser beam was used to irradiate a nanoparticle embedded in the bilayer that immediately induced local melting when the temperature exceeded the melting transition, *T*_*m*_* = *33.8 °C, of the bilayer^25^. Upon melting, the phase sensitive fluorescent molecules partitioned into the melted region producing a fluorescent footprint. In Fig. 2c, representative images of the melted footprints for all three nanoparticles at the same laser intensity are shown. The melted footprints are clearly seen as bright fluorescent circles centered at the position of the nanoparticle, the largest footprint was induced by the AuNSs and the smallest by the 80 nm AuNPs. The radial size, *D*_*m*_, of the melted footprints increased with laser intensity (see Supplementary Fig. S2) while for all the individual laser intensities the temperature at the phase boundary equaled *T*_m_ which allowed calculation of the constant, *C*, in equation (1).

By inserting the experimental value for *C* and the radius of the nanoparticles in equation (1), the surface temperature as a function of laser intensity was found for all three nanoparticles, see Fig. 2d. The surface temperatures of all three nanoparticles increased linearly with laser power as predicted by equation (1), though, for the highest laser intensity used to irradiate the AuNSs, the temperature increase saturated around 240 °C. This temperature saturation is in accordance with reported observations of temperature profiles of irradiated metallic nanoparticles^16^,^46^ suggesting that a temperature induced phenomenon, possibly micro-bubble generation, may kick in at the surface of continuous wave irradiated nanoparticles reaching temperatures beyond 240 °C. Another possibility could be that the AuNSs disintegrate upon extensive heating, however, if we reheated AuNSs after allowing the bilayer to equilibrate to gel phase, it was in most cases possible to induce a melting footprint once more, thus indicating that the AuNSs are still able to heat its surroundings and probably were not damaged (see Supplementary Fig. S3). While the temperature increase of the AuNPs, in agreement with literature, increases with particle size^16^,^45^, the heat generation of the AuNSs was substantially higher than of any of the AuNPs at all laser intensities. The heat conversion efficiency for each nanoparticle can be derived from linear fits to the data in Fig. 2d, yielding (1412 ± 800.8) K/W for the AuNSs, (573.7 ± 33) K/W for 150 nm AuNPs, and (492.3 ± 80) K/W for the 80 nm AuNPs (errors given by the 95% confidence interval).

While the single particle surface temperatures are of interest for some applications, we note that particle surface temperatures alone cannot be used for predicting the bulk heating efficiency of different types of nanoparticles, as this depends on the geometry of the nanoparticles in the solution^47^ and on the nanoparticles’ absorption cross-section. Our assay provides an experimental quantification of the absorption cross-section by using the following equation^47^


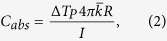


where Δ*T*_*P*_ is the nanoparticle’s surface temperature, 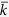 is the heat conductivity of the combined glass and water system at 300 K which we approximate to be a simple average of *k*_*glass*_ = 1.1 W/m K^48^ and *k*_*water*_ = 0.61 W/m K as suggested in ref. 47, *I* is the laser intensity and *R* is the particle radius. Using equation (2) we get *C*_*abs*_ = (5781 ± 711) nm^2^ for a AuNS, (1332 ± 399) nm^2^ for a 150 nm AuNP, and (390 ± 138) nm^2^ for a 80 nm AuNP. These numbers can be compared to the theoretical values which can be obtained from Supplementary Fig. S1: 2990 nm^2^ for a AuNS, 984 nm^2^ for a 150 nm AuNP and 98 nm^2^ for a 80 nm AuNP. The experimental values are found to be higher than the theoretically calculated values even when including the uncertainties. This discrepancy between theory and experiments may originate from uncertainties in determining the laser intensity at the position of the nanoparticle as well as uncertainties in determining the combined heat conductivity of the water-glass system experienced by a particle which has a point contact with the glass, but is immersed in the water.

### Quantifying bulk heating *in vitro*

**Figure 3:**
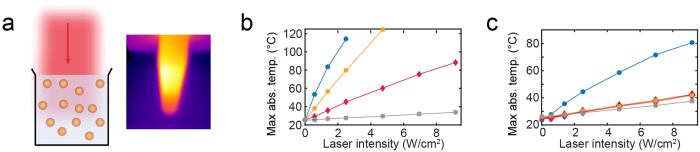
Thermographic assessment of the heat generation *in vitro*. (**a**) Illustration of the assay using thermographic imaging to assess the laser mediated bulk heating. (**b,c**) Plots of the maximum absolute temperature in aqueous bulk solutions as a function of laser intensity measured in (**b**) 5 × 10^10^ nanoparticle/mL solutions or (**c**) 5 × 10^8^ nanoparticle/mL solutions of AuNSs (blue circle), 80 nm AuNPs (red diamonds), 150 nm AuNPs (yellow squares), and saline (grey stars). The laser intensity was 0.58 W/cm^2^.

To analyze the heating capabilities of the nanoparticles in bulk, we irradiated aqueous solutions of the nanoparticles with the 807 nm diode laser and measured the bulk temperature using thermographic imaging every 15 seconds (see sketch in Fig. 3a). First, we prepared nanoparticle solutions of 5 × 10^10^ nanoparticles/mL (same concentration was used for all nanoparticle types). The maximum temperature in the solution for each laser intensity is shown in Fig. 3b.

The temperature in the AuNS solution rapidly increased and exceeded 100 °C at a laser intensity of *P* ~2 W/cm^2^, the 150 nm AuNP solution reached 100 °C at a laser intensity of *P* ~4 W/cm^2^, and the 80 nm AuNP solution only reached ~80 °C for the highest laser intensity used. The saline solution only heated slightly, reaching a temperature increase of Δ*T*~10 °C for the highest laser intensity used. To investigate the effect of the nanoparticle concentration, we repeated the experiment in 100-fold dilutions (see Fig. 3c). Here, we only observed a temperature increase distinct from the saline sample in the AuNS solution; however, it was significantly smaller than observed in the high concentration sample. These concentration-dependent observations are in agreement with findings in literature^35^,^49^.

### Evaluating the photothermal treatment response *in vivo*

^18^F-FDG PET imaging of mice bearing subcutaneous human neuroendocrine lung carcinoid (H727) tumor xenografts was used to compare the photothermal effect of the nanoparticles *in vivo*. When the tumor volume reached ~700 mm^3^ the animals were divided into four groups receiving the same number of nanoparticles of the various types tested (50 μL of 5 × 10^10^ nanoparticles/mL of 80 nm AuNPs, 150 nm AuNPs, AuNSs). In addition, we also had a control group receiving 50 μL of saline (*n* = 6, each group). As we in this study focused on the heat-conversion efficiencies of the different nanoparticles it was important to have a fixed number of nanoparticles in the tumor. To obtain this, the same concentration of nanoparticles was delivered by intratumoral injection in all experiments. Immediately after the injection, the mice were irradiated with the diode laser (807 nm) for 5 minutes at the tumor region with a laser intensity of 0.58 W/cm^2^. In addition, control groups of sham treated mice receiving nanoparticles but no irradiation were included (*n* = 3, each group). Thermographic imaging every 15 s was used to assess the temperature increase at the surface of tumor xenografts in the different treatment groups while they were irradiated and all mice were ^18^F-FDG PET scanned one day prior to the treatment (Baseline), an hour after (Day 0) and at 2 days (Day 2) after laser irradiation.

**Figure 4:**
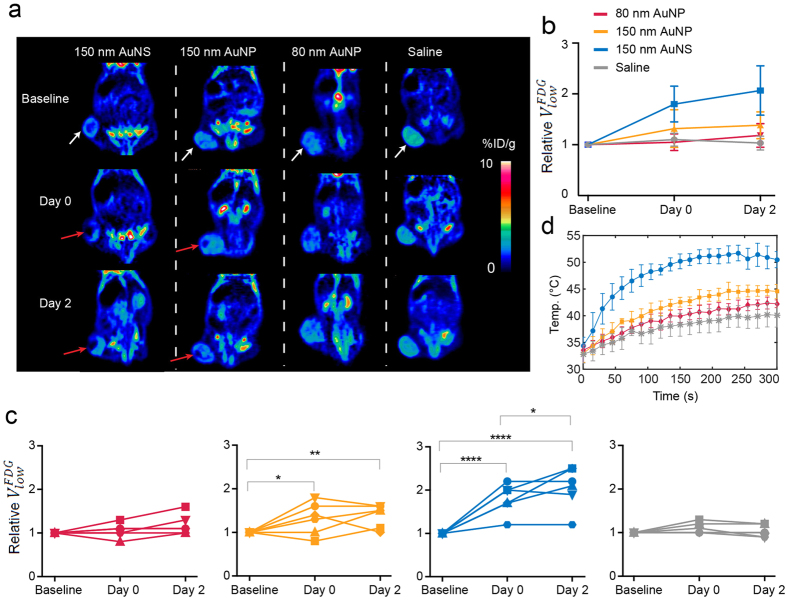
PET evaluation of the treatment response. (**a**) Images showing the coronal planes of the ^18^F-FDG PET scan of mice from each nanoparticle group (columns). The rows show the baseline PET scan, the scan immediately after the laser irradiation (Day 0), and the scan two days post-treatment (Day 2). White arrows mark the tumor location where the nanoparticles were administered and subsequently irradiated with the 807 nm laser (laser intensity of 0.58 W/cm^2^). In the mice from the AuNS and 150 nm AuNP groups there is a tumor subvolume with decreased uptake of ^18^F-FDG (marked by red arrows) both at Day 0 and Day 2. (**b,c**) 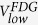 at different timepoints. If the treatment is effective this volume will increase after laser irradiation. Each individual data set has been normalized to the baseline value. The mean relative changes in the tumor volume with low ^18^F-FDG uptake in the four treatment groups are shown in (**b**) and for individual animals in (**c**) (red: 80 nm AuNP, yellow: 150 nm AuNP, blue: 150 nm AuNS, and grey: saline). *n = *6, each group. (**d**) Plots of the temperature evolution measured using thermographic imaging at the skin surface of the tumors of mice receiving AuNSs (blue circle, *n* = 3), 80 nm AuNP (red diamonds, *n* = 3), 150 nm AuNP (yellow squares, *n* = 3), and saline (grey stars, *n* = 7). The laser intensity was 0.58 W/cm^2^. Error bars represent one SD. * denotes p value < 0.05, ** denotes p value < 0.01, and **** denotes p value < 0.0001.

Figure 4a shows representative coronal images of the whole-body ^18^F-FDG uptake in mice from the four treatment groups at Baseline, Day 0, and Day 2 scan. By visual inspection of the PET images it was evident that mice from the AuNS group generally had a subvolume in the treated tumor area with low or no uptake of ^18^F-FDG, both immediately and two days after treatment. The same was also the case for some mice in the 150 nm AuNP group, while other showed no clear visual effect. Contrary, no changes in ^18^F-FDG uptake over time were observed in mice from the 80 nm AuNP and saline groups. The signal loss occurring post-treatment is a strong marker that the cancer cells in this region have reduced metabolism or are ablated as a consequence of the treatment (region is marked by red arrows in Fig. 4a). No visual change in ^18^F-FDG tumor uptake was observed in any of the sham treated animals as a result of nanoparticle administration (See Supplementary Fig. S4).

To quantify ^18^F-FDG accumulation we defined regions of interest (ROIs) on whole tumor regions and extracted the uptake as percent of injected dose per grams of body weight (%ID/g). The different treatment groups had comparable mean and maximum tumor uptake of ^18^F-FDG (see Supplementary Fig. S5). However, from the PET images it was clear that the treatment was limited to a rather small tumor subvolume. Therefore, mean and maximum ^18^F-FDG uptake that is commonly used for treatment response evaluation, is inexpedient for quantifying the effect of heating in this setup. Instead, to quantify the tumor volume with low or no uptake of ^18^F-FDG, we determined the tumor volume in which the intensity was lower than the mean of the distribution of %ID/g minus 3/2 of the standard deviations. This volume is denoted 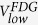 and will increase after laser irradiation if the treatment generates sufficient heat to damage cancer cells. As the size of 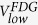 can be influenced by other factors, especially the degree of tumor necrosis, each individual data set was normalized to the value from the baseline scan in order to assess the relative change after treatment. In Fig. 4b,c the mean and individual relative changes in 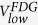 is shown for each group. After treatment, the mean 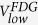 in the AuNS group increased significantly over time and the relative change was also significantly larger than in all other groups both at Day 0 and Day 2, (p value < 0.005 on Day 0 and p value < 0.0001 on Day 2 compared to 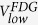 of the other groups). Contrary, the relative changes in mean 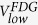 of the 80 and 150 nm AuNP groups were not significantly different from the saline group on either Day 0 or Day 2. When looking at individual animals in the AuNS group, five animals had an increase in 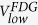 after treatment, while one mouse showed an unaltered volume comparable to animals in the saline group. In the 150 nm AuNP group only some of the mice had a treatment response, overall leading to a small but significant mean increase in 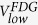 after treatment. No change in 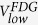 was observed over time in the 80 nm AuNP experiments nor in the saline group or in the sham treated animals (see Supplementary Fig. S6), confirming that a combination of nanoparticle injection and irradiation is required to induce a change in ^18^F-FDG tumor uptake.

Further, thermographic imaging (see Fig. 4d; *n* = 3, each group) showed that the maximum temperature at the surface of the AuNS laden tumors increased rapidly upon laser irradiation where after it reached a plateau (~after 3 min) with a maximum temperature of 51.7 ± 3.4 °C. In the other three groups the temperature of the tumor area increased more slowly throughout the entire duration of laser irradiation. The highest temperature measured was (42.4 ± 1.7) °C in the 80 nm AuNP group, (44.7 ± 1.7) °C in the 150 nm AuNP group, and (40.2 ± 2.4) °C in the saline group. These measurements support the findings from the PET-based evaluation, and are a clear indication that higher temperatures were reached in the mice receiving AuNSs compared to the other groups.

## Discussion

In recent years much effort has been devoted to developing new nanoparticles for photothermal cancer therapy. There are different factors that determine their potential; *e.g.,* their capabilities as light-to-heat converters when irradiated with laser light capable of penetrating tissue, their tumor accumulation, and their stability and toxicity. As different experimental conditions affect treatment outcome, it is often difficult to compare nanoparticles between studies in literature. Here we used different methods to compare the heat generation of nanoparticles for photothermal cancer therapy.

At the single particle level our theoretical FEM calculations (Supplementary Fig. S1) and *in vitro* direct measurement allowed us to assess the particle temperature (Fig. 2d) and the absorption cross-section. As expected, the resonant AuNSs performed substantially better as photothermal heat transducers if irradiated by NIR light compared to the non-resonant AuNPs. Interestingly, the temperature differences between the nanoparticles measured in bulk were even more significant as shown in Fig. 3d. This is explained by the fact that the AuNSs have a significantly larger absorption cross-section than the AuNPs. This effect is also clearly observed in Fig. 2c and Supplementary Fig. S2 where the larger nanoparticles, with the highest absorption cross-section, produce significantly greater melted footprints compared to the 80 nm AuNPs. Limitations to the *in vitro* setup relates to when the extent of the melted footprint is so small that it is comparable with the diffraction limit of the imaging laser light (λ = 488 nm), leading to slightly overestimated surface temperatures (see footprints of the 80 nm AuNPs in Fig. 2d and Supplementary Fig. S2). Also, the small melted footprints for this nanoparticle size are mostly confined within the NIR laser focus and additional background heating on the order of 1K is expected to occur^50^.

*In vivo*, PET imaging of tumor uptake of ^18^F-FDG was used to compare the effect of heating in nanoparticle-laden subcutaneous human tumor xenografts in mice (Fig. 4), and the results agreed with the findings from the *in vitro* assays. In the field of nanoparticle-mediated photothermal cancer therapy, PET has mainly been used to track and localize radiolabeled nanoparticles^51^,^52^. However, PET imaging also holds a great potential for early evaluation of treatment response and can potentially improve treatment outcome, as well as reduce the time and cost. ^18^F-FDG, the most widely used PET tracer in oncology, has been particularly successful due to tumor cells’ increased glucose metabolism, and it has been used clinically for both tumor diagnosing and evaluation of treatment response^53^,^54^. ^18^F-FDG PET imaging has previously also been applied to quantify the effect of photothermal cancer therapy in a few preclinical studies^9^,^12^ and overall our results support the use of ^18^F-FDG PET as a reliable and fast evaluation tool for the treatment effect of nanoparticle-based photothermal cancer therapy. Limitations to the *in vivo* setup include variations in the deposition of the nanoparticles during intratumoral injection, which can affect the effect of heating. Small differences in the depth of the intratumoral injection and back leakage of nanoparticles due to high interstitial pressure are likely to have caused some of the variation observed in the treatment response within each group. Finally, because necrotic tissue has no ^18^F-FDG uptake, the degree of necrosis within a tumor can affect the size of 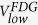. Therefore, it is important that the tumor size within each group is matched.

In summary, the *in vitro* screening assay has the great advantage that it is fast and easy to perform and reliably pinpoints which nanoparticles have potential for photothermal therapy. This screening can be performed before selecting and optimizing nanoparticles for *in vivo* conditions and introducing the nanoparticles into animal models, thereby reducing the number of animals needed for the *in vivo* evaluation. However, although the *in vitro* assay reliably pinpoints which nanoparticles are most efficient light-to-heat converters, there are other factors in a biological context that are not accounted for in an *in vitro* system, e.g., delivery efficiency, particle-coupling effects^11^,^55^,^56^, or particle disintegration. Hence, the *in vitro* assay cannot stand alone and should be accompanied by *in vivo* evaluation of treatment response. Our results from photothermal therapy of tumor-bearing mice shows that PET imaging can provide a quantitative measure of how efficient different nanoparticles are at killing cancer cells. This is achievable on a time-scale of hours after treatment where standard evaluation methods based on morphological changes cannot yet detect any treatment response. We propose to use the presented strategy to assess and improve the potential for photothermal therapy of any type of nanostructure, while reducing time, cost, and number of animals. It is our hope that these methods will be systematically used as a platform for efficient testing of the plethora of new emerging plasmonic nanoparticles being developed for photothermal cancer therapy.

## Methods

### Physical properties of the nanoparticles

The AuNSs (NanoComposix, USA) consisted of a 119.7 ± 8.9 nm dielectric silica core surrounded by a thin gold shell (total diameter of 151.3 nm ± 7.7 nm; measured by supplier using transmission electron microscopy (TEM)). The AuNPs were spherical solid gold nanoparticles with diameters of 150 nm (Nanopartz, USA; diameter: 150 nm ± 6 nm measured by supplier using TEM) and of 80 nm (NanoComposix USA; diameter: 81.7 nm ± 11.4 nm measured by supplier using TEM). All nanoparticles were obtained functionalized with 5 kDa poly(ethylene glycol) (PEG). Upon arrival, the UV-vis spectra of the nanoparticles were measured in aqueous solutions using spectrophotometry (Fig. 2b) and the hydrodynamic diameters and zeta-potentials were measured using Zetasizer Nano Series (Supplementary Table 1). The theoretical absorption cross-sections were calculated by building a 3D simulation model using COMSOL multiphysics, which is a commercially available FEM package (see Supplementary Methods and Supplementary Fig. 1).

### *In vitro* 2D heating assay

The phospholipid bilayer was prepared by mixing 2 μmoles of saturated lipids (1,2-dipentadecanyol-sn-glycero-3-phosphicholine), with 1 mole% of 3,3′-dilinoleyloxacarbocyanine perchlorate (*FAST*-DiO) fluorophores and chloroform. In a clean vial, a thin film of the lipid-dye mixture was carefully evaporated under nitrogen flow and left in vacuum for 2 hours. 1 mL of preheated 150 mM NaCl PBS buffer was added to hydrate the lipid-dye film and left for 1 hour at 37 °C. After hydration, the mixture was extruded through polycarbonate filters with 50 nm pore size to create small unilamellar vesicles. To create a highly hydrophilic glass surface to support the lipid bilayer, clean glass slides were O_2_ plasma cleaned for at least 30 min. A sample chamber was constructed and 200 μL of the extruded unilamellar vesicles were added to fuse with the glass surface and incubated for 2 hours at 37 °C. Excess vesicles were removed by thoroughly washing the supported phospholipid bilayer with milliQ water preheated to ~50 °C. Nanoparticles suspended in milliQ water were added and allowed to adhere to the bilayer. Only nanoparticles that were stuck to the bilayer were used for experiments.

The experiments were conducted on a Leica SP5 confocal microscope equipped with a piezoelectric stage into which an optical trap based on a 1064 nm tightly focused Gaussian beam was implemented (see beam profile in Supplementary Fig. 7a)^57^. A 100 × 1.4 NA Leica apochromatic oil objective was used to focus the optical trap and for visualization. The nanoparticles were imaged in reflection mode (excited by λ = 594 nm). *FAST*-DiO was excited by λ = 488 nm and emission collected with a bandwidth from 496 nm to 587 nm. The ambient temperature in the sample chamber was kept constant by using a thermostated stage and was measured to be *T*_*ambient*_ = 26.7 °C by inserting a temperature probe into the chamber after the experiments. This value was used for the data analysis of the *in vitro* experiments.

### Image analysis in the *in vitro* study

Images were analyzed in Matlab. The size of the individual melting footprint centered around the nanoparticle equals the distance from the particle center (which was located in every image by a centroid algorithm) to the location of the melting transition (*D*_m_) where the temperature was *T*_m_(*D*_m_) = 33.8 °C^25^. First, the symmetry of the melting footprints was exploited by performing a rotationally averaged radial intensity plot (averaged from 0 to 2π for every distance from the nanoparticle). Subsequently, *D*_m_ was quantified by applying a threshold, *Tr*, to the images to distinguish the melted zone, containing higher density of fluorophores, from the background which consisted of a gel phase bilayer with a lower density of fluorophores. The background was quantified as the average of a region of interest, ROI_bg_, selected several micrometers away from the melted zone and the threshold was defined to be the intensity value at which the intensity had dropped to 50% of the maximum value inside a region of interest of the melted region (ROI_melted_): 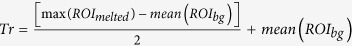. To find the temperature at the surface of the nanoparticle (D_*p*_ = *R*) we used equation (1) to relate the temperature elevations at the nanoparticle surface and at the boundary of the melting region: Δ*T*(D*p*) = Δ*T*(D_*m*_)D_*m*_/D_*p*_ where Δ*T*(D_*m*_), D_*m*_ and D_*p*_ were experimentally measured.

### Thermographic assessment of the photothermal efficiency in bulk and *in vivo*

500 μL aqueous solutions of all nanoparticles were prepared in Eppendorf tubes (concentrations of 5 × 10^10^ nanoparticles/mL and of 5 × 10^8^ nanoparticles/mL). Each nanoparticle solution and saline solution were irradiated continuously from the side with the 807 nm diode laser (beam diameter: ~1 cm) while the laser intensity was increased every 5 min (in the interval from 0 W/cm^2^ to 9.2 W/cm^2^). Real-time imaging of the sample temperature was recorded with a FLIR NIR thermal camera every 15 s. The images were analyzed using FLIR tools software to infer the maximum temperature in the sample.

In addition, during the 5 min laser irradiation of the tumor bearing mice, real-time thermographs were also taken and analyzed following the same procedure as in the *in vitro* bulk measurements.

### Animal model

All animal experiments have been approved by the Danish Animal Welfare Council, ministry of Justice. All methods and experiments were carried out in accordance with the approved guidelines. Upon arrival the mice were allowed to acclimatize for one week in the animal facility and had access to water and chow ad libitum. Human neuroendocrine lung carcinoid cell line H727 (ECCAC, Salibury, UK) were cultured in standard medium (RPMI medium 1640 + GlutaMAX) supplemented with 10% fetal calf serum and 1% penicillin-streptomycin at 37 °C and in 5% CO_2_. Tumor xenografts were established by subcutaneous injection of ~10^6^ H727 cells, dissolved in 100 μl growth medium mixed with 100 μl matrigel, into the left flank of the 6 weeks old female NMRI nude mice. Tumor dimensions were measured with a caliper on a regular basis and the volume calculated as volume = ½(length × width^2^). When tumors reached ~700 mm^3^ animals were matched into four treatment groups based on their tumor volumes; 80 nm AuNPs: 698.5 mm^3^ (mean) ± 144.7 mm^3^ (±SD), 150 nm AuNPs: 673.4 mm^3^ ± 191.2 mm^3^, AuNSs: 699 mm^3^ ± 234.3 mm^3^, or saline: 683.2 mm^3^ ± 203.6 mm^3^, (n = 6, each group). Three sham treatment groups were included (AuNS sham: 503.8 mm^3^ ± 298.9 mm^3^, 150 nm AuNP sham: 349.8 mm^3^ ± 142.7 mm^3^, and 80 nm AuNP sham: 375.6 mm^3^ ± 205.7 mm^3^) (n = 3, each group).

### *In vivo* photothermal therapy

During treatment the animals were anesthetized by sevoflurane and their temperature kept stable. The mice were injected approximately 3 mm into the tumor with 50 μl of 5 × 10^10^ nanoparticles/mL or 50 μl saline using a programmable syringe pump with a flow rate of 5 μl/min (1 ml syringe, 25G needle). After injection the needle was left in the tumor for 10 min to equalize pressure and prevent back flow of blood and nanoparticles. Immediately after, the mice were irradiated for 5 min with a laser power density of 0.58 W/cm^2^ with an 807 nm diode laser. This laser power was decided upon as it was the highest power we could use without inflicting burns and scab formations. The diode laser was collimated with a beam diameter of 10.72 mm (see Supplementary Fig. 7b). Two days after the photothermal treatment, the mice were euthanized.

### PET/CT imaging

The mice were PET scanned the day before (baseline), 1 h (Day 0), and two days (Day 2) after treatment. ^18^F-FDG was obtained at Department of Nuclear Medicine & PET, Rigshospitalet, Denmark. The animals were anesthetized by sevoflurane and their temperature kept stable. Mice were injected with ~10 MBq ^18^F-FDG through a tail vein, and PET scanned for 10 min 1 hour post injection using a small animal PET scanner. The energy window of the emission scans was set to 350–650 KeV with a time resolution of 6 ns. Acquired datasets were stored in listmode, post processed into 128 × 144 × 95 sinograms, and reconstructed using maximum *a posteriori* (MAP) algorithm into 256 × 256 × 95 matrixes with a voxel size of 0.29 × 0.29 × 0.79 mm^3^ and a resolution of 1.2 mm at center field of view. Emission data was corrected for dead time and decay. Each PET scan was followed by a CT scan. ROIs were drawn on whole tumor regions and uptake quantified as mean and maximum %ID/g. Moreover, a threshold of the mean tumor uptake minus 3/2 standard deviations was applied to quantify the tumor volume with low or no ^18^F-FDG accumulation in all PET images 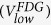. Each individual data set was normalized to the baseline value and the relative change in this volume within each group calculated as mean ± SD.

### Statistics

Groups in the *in vivo* experiments were compared using a two-way ANOVA with repeated measures and Tukey’s post hoc test. Statistical analysis was performed in GraphPad Prism 6 and *P* < 0.05 was considered as statistical significant in all analyses.

## Additional Information

**How to cite this article**: Jørgensen, J. T. *et al*. Single Particle and PET-based Platform for Identifying Optimal Plasmonic Nano-Heaters for Photothermal Cancer Therapy. *Sci. Rep.* **6**, 30076; doi: 10.1038/srep30076 (2016).

## Supplementary Material

Supplementary Information
