# Supplementary material for: Single Particle and PET-based Platform for Identifying Optimal Plasmonic Nano-Heaters for Photothermal Cancer Therapy
Source: Sci Rep. 2016 Aug 2;6:30076. doi: 10.1038/srep30076 (PMC4969617; doi:10.1038/srep30076)
Supplement: Supplementary Information [file srep30076-s1.pdf]

Supplementary Information to:

Single Particle and PET-based Platform for Identifying Optimal Plasmonic Nano-Heaters for Photothermal Cancer Therapy

Jesper Tranekjær Jørgensen[1]*, Kamilla Norregaard[1,2] *, Pengfei Tian[2,3], Poul Martin Bendix[2], Andreas Kjaer[1], Lene B. Oddershede[2,§]

*These authors contributed equally to this work

[§]Correspondence should be addressed to email:oddershede@nbi.ku.dk

[1]Dept. of Clinical Physiology, Nuclear Medicine & PET and Cluster for Molecular Imaging, Rigshospitalet and University of Copenhagen, Denmark
[2]Niels Bohr Institute, University of Copenhagen, Denmark
[3]Laboratory of Chemical Physics, National Institute of Diabetes and Digestive and Kidney Diseases, National Institutes of Health, Bethesda, Maryland, United States.

Supplementary Methods

Theoretical model of photothermal effect of silica-gold nanoshells and colloidal gold nanoparticles

A plasmonic model is established to resolve the resistive heat generated by the metallic nanostructure during a photothermal process. The equation that describes the electromagnetic field of the metallic absorber subject to the irradiating laser power of angular frequency ω, is written as:

$$\nabla \times \mu_r^{-1}(\nabla \times E(r)) - K_0^{\,2}(\varepsilon_r(r) - j\sigma/\omega\varepsilon_0)E(r) = 0, \quad (1)$$

where E is the position *(r)* dependent electric field, ε_r and ε_0 are the relative permittivity and the permittivity of vacuum respectively, μ_r represents the relative permeability, K_0 and σ are the free space wavenumber and electrical conductivity respectively. The electric field E and the relative permittivity ε_r are computed by solving equation S(1) using FEM method[1–3], whose essential idea is to obtain the numerical solution by, first, discretize the region of interest into small elements (see Figure S1b), and then approximate the solution over each element by a simple interpolation function. Thereby the absorption power per unit volume (see Figure S1a) is obtained as:

$$Q_{abs}(r) = \frac{\omega\varepsilon_0}{2} Im\{\varepsilon_r(r)\}|E(r)|^2, \quad (2)$$

from which the absorption cross section C_{abs} is given by:

$$C_{abs} = \frac{1}{I}\int Q_{abs}(r)dr, \quad (3)$$

where I is the light intensity. Taking into account the geometry of the structure, the 3D simulation model is built using COMSOL multiphysics. The AuNS nanostructure is modeled as concentric spheres of radii 50 nm and 75 nm as shown in Figure S1c. The nanostructures (AuNSs and AuNPs) are put in the center of a spherical domain of water (with a radius of 1 µm), and then surrounded by an absorbing perfectly matched layer (500 nm in thickness) which could minimize undesirable reflection of the electromagnetic

wave when passing through the artificial boundary. In order to ensure the simulation is well converged, the total number of iterations was chosen so that the relative tolerance of electric field *E* is better than 0.1%. The optical constant of gold used here is referred to the measurements from the previous experiments[4].

Supplementary References

1. Chen, X., Chen, Y., Yan, M. & Qiu, M. Nanosecond photothermal effects in plasmonic nanostructures. *ACS Nano* **6,** 2550–2557 (2012).

2. Ma, H., Tian, P., Pello, J., Bendix, P. M. & Oddershede, L. B. Heat generation by irradiated complex composite nanostructures. *Nano Lett.* **14,** 612–9 (2014).

3. Knight, M. W. & Halas, N. J. Nanoshells to nanoeggs to nanocups: optical properties of reduced symmetry core–shell nanoparticles beyond the quasistatic limit. *New J. Phys.* **10,** 105006 (2008).

4. Johnson, P. B. & Christy, R. W. Optical Constants of the Noble Metals. *Phys. Rev. B* **6,** 4370–4379 (1972).

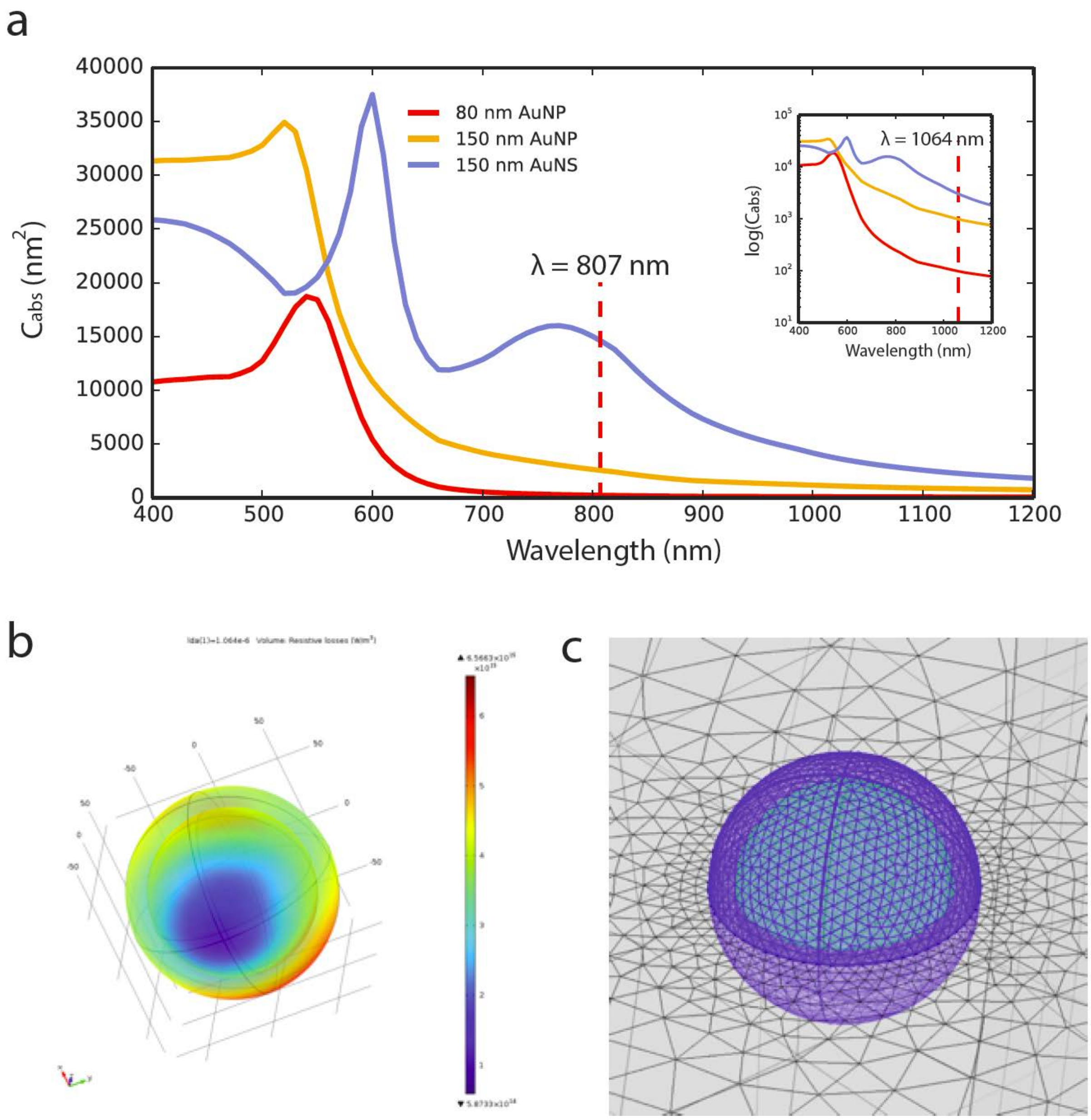

Supplementary Figure S1: Finite Element Method calculations. (a) The absorption cross-sections (C_{abs}) for each of the nanoparticles are calculated as a function of wavelength. The wavelength marked by the dotted line represents the *in vivo* laser at λ=807 nm. *Inset*: C_{abs} plotted on a semi-log scale to better resolve the C_{abs} values at wavelength λ=1064 nm (*in vitro* laser wavelength; marked by a dotted line). Throughout the entire NIR region, C_{abs} of the AuNS are significantly larger than that of the AuNPs. (b) Simulated distribution of absorption power of a 150 nm AuNS under laser irradiation with the light intensity of 1 mW/μm^2 and wavelength of 1064 nm. (c) Finite element meshing scheme of a simulated AuNS by the FEM method, blue is the gold shell and green is the silica core, generated by COMSOL.

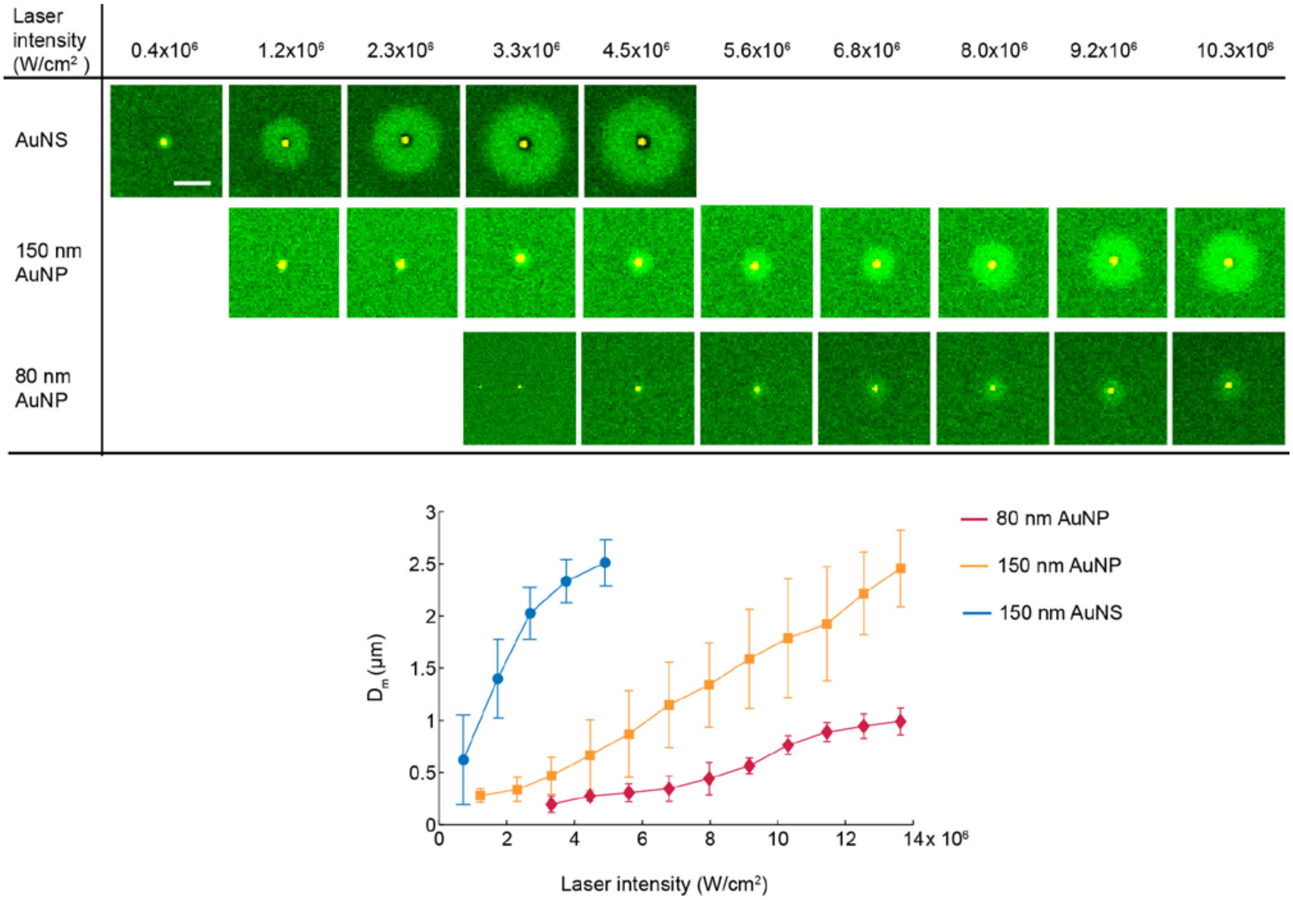

Supplementary Figure S2: 2D *in vitro* heating experiments. The heat generation of a single nanoparticle is shown as a function of laser intensity (columns) for each of the nanoparticles (rows). The size of the melted footprint increases with laser intensity. The scalebar is 2 µm and is the same in all images. Below is shown the analyzed diameter, D_m, of the melted footprints for all three nanoparticles as a function of laser intensity.

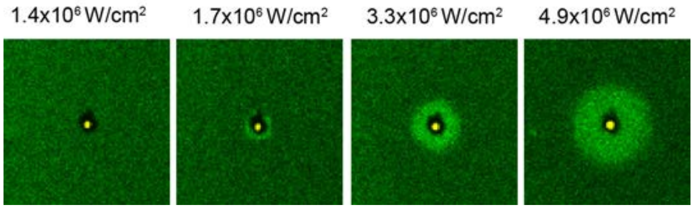

Supplementary Figure S3: Re-heating of an AuNS embedded in the *in vitro* 2D lipid bilayer. The lipid bilayer in the immediate vicinity of the AuNS appears black in the image as it has been damaged in a previous heating series of this AuNS. When the laser intensity exceeds 1.7x10^6 W/cm^2 the temperature profile of the AuNS becomes large enough to induce melting of the undamaged bilayer seen by the intensity increase of the fluorescent molecules in this and the subsequent images with increasing laser intensities.

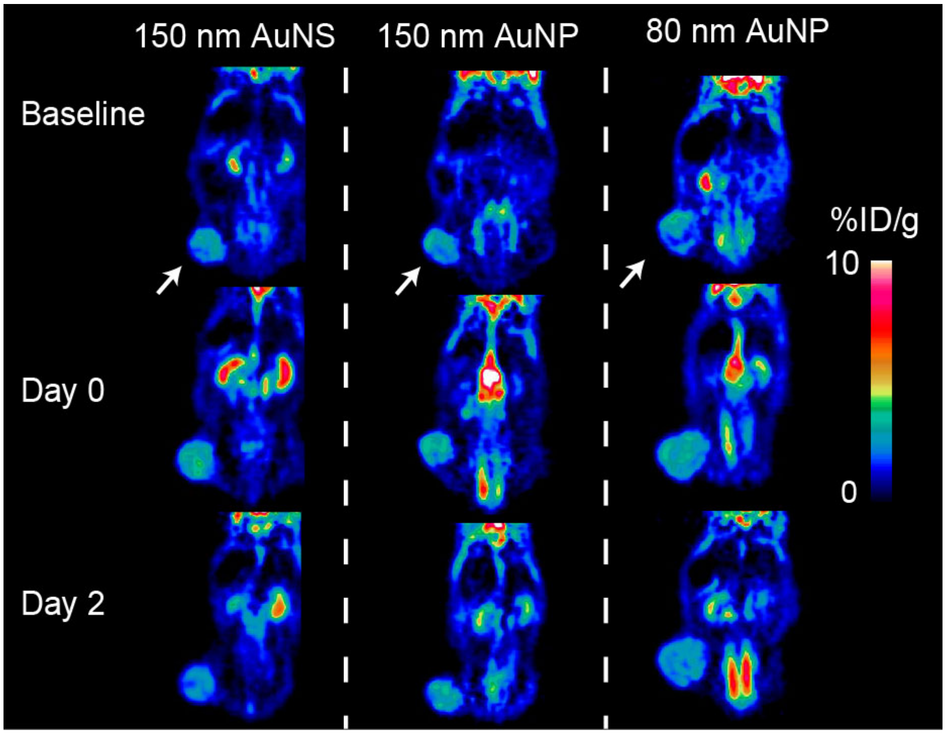

Supplementary Figure S4: Representative ^{18}FDG PET images of sham treated mice (i.e., received nanoparticles but no laser irradiation). Images showing the coronal planes of the ^{18}F-FDG PET scan of sham treated mice from each nanoparticle group (columns). The rows show the baseline PET scan, the scan immediately after the sham treatment (Day 0), and the scan two days post-treatment (Day 2). White arrows mark the tumor location where the nanoparticles were administered.

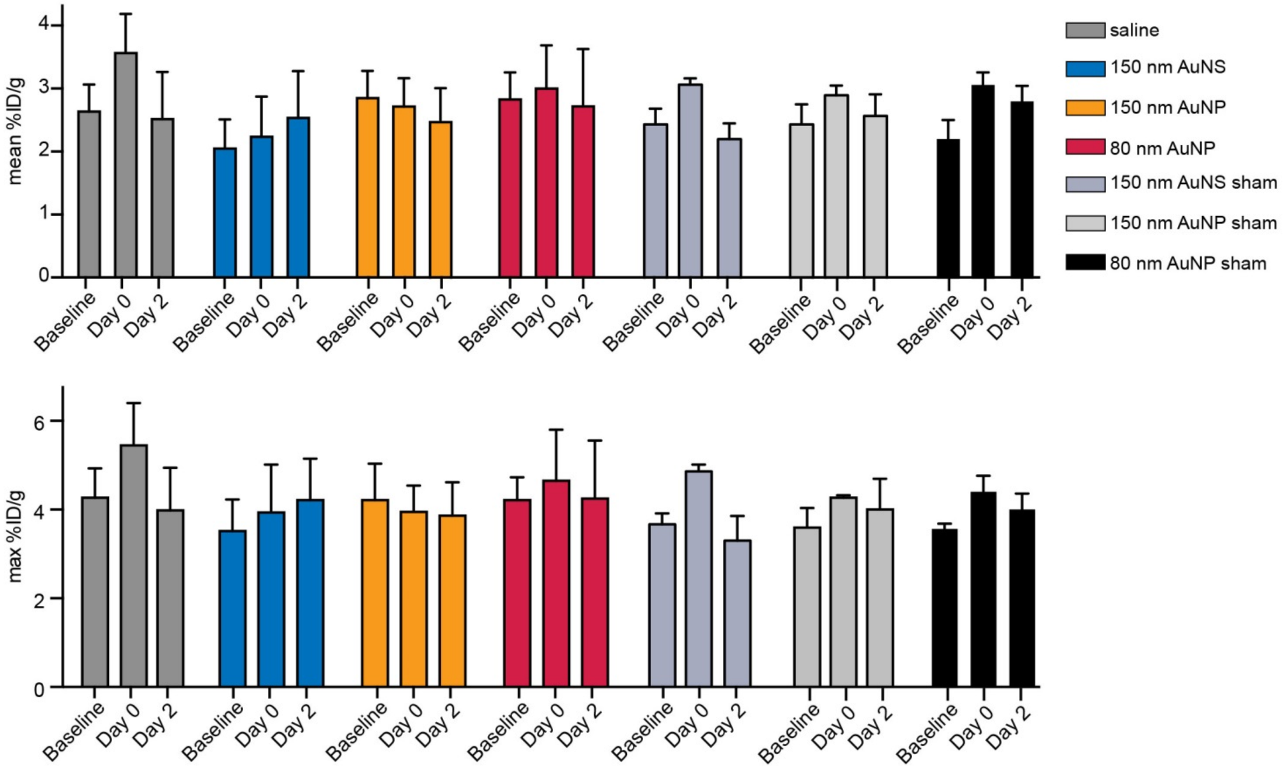

Supplementary Figure S5: Mean and maximum (%ID/g) ^{18}F-FDG uptake in the tumors. PET scan was acquired prior to photothermal treatment (Baseline), approx. one hour after the treatment (Day 0), and two days after the treatment (Day 2). *n* = 6 in each group, errorbars represent SD. No significant decrease in mean and max %ID/g after treatment was found in any of the groups.

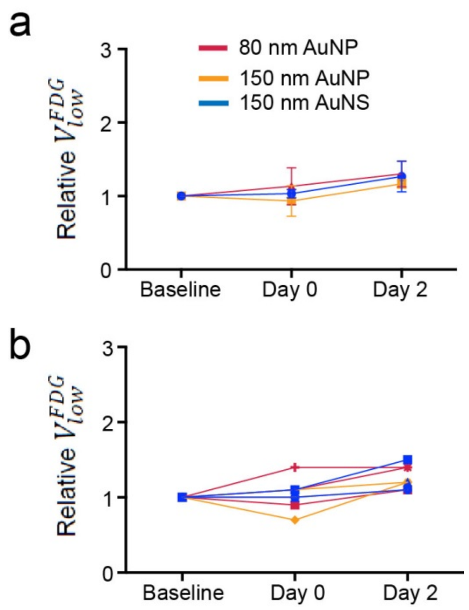

Supplementary Figure S6: Sham treated mice (i.e., received nanoparticles but no laser irradiation). (a) The mean V_{low}^{FDG} relative to the baseline value (*n* = 3, each group and the errorbars represent SD). The lack of increase in V_{low}^{FDG} indicates that the nanoparticles have no toxic or cell damaging effect. (b) V_{low}^{FDG} relative to the baseline value for all individual animals. There is no statistical different between the sham treated groups and the saline receiving group.

Supplementary Table S1: Dynamic light scattering and surface charge measurements of PEG-functionalized nanoparticle suspended in MilliQ water.

	80 nm AuNP	150 nm AuNP	150 nm AuNS
Hydrodynamic diameter (nm)	113.3 ± 0.8	155.9 ± 2.3	198 ± 1.2
Z-potential (mV)	-27 ± 0.7	-34.9 ± 1.1	-36.5 ± 0.6

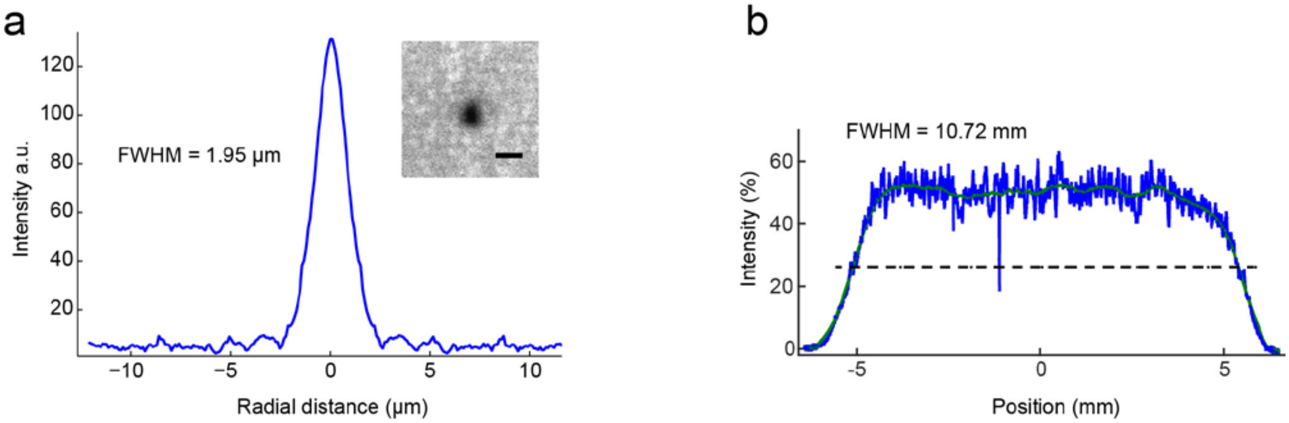

Supplementary Figure S7: Laser profiles of the *in vitro* (λ=1064 nm) and *in vivo* (λ=807 nm) lasers. (a) The laser profile of the 1064 nm *in vitro* laser was mapped out by bleaching an Alexa555 fluorophore-labeled bovine serum albumin layer supported by a microscope coverslip (image shown in *inset*). The FWHM was quantified yielding a focus area of $2.99x10^{-12}$ m^2. Scale bar in *inset* is 2 µm. (b) The laser profile of the 807 nm *in vivo* diode laser was mapped out by translating a beam profiler through the focus of the laser beam. The FWHM was quantified yielding a focus area of 0.9 cm^2.
